# Lipid–Polymer Hybrid Nanoparticles in Microparticle-Based Powder: Evaluating the Potential of Methylprednisolone Delivery for Future Lung Disease Treatment via Inhalation

**DOI:** 10.3390/pharmaceutics16111454

**Published:** 2024-11-14

**Authors:** Cinzia Scialabba, Emanuela Fabiola Craparo, Sofia Bonsignore, Marta Cabibbo, Gennara Cavallaro

**Affiliations:** Laboratory of Biocompatible Polymers, Department of Biological, Chemical and Pharmaceutical Sciences and Technologies (STEBICEF), University of Palermo, Via Archirafi 32, 90123 Palermo, Italy; cinzia.scialabba@unipa.it (C.S.); sofia.bonsignore@unipa.it (S.B.); marta.cabibbo@unipa.it (M.C.); gennara.cavallaro@unipa.it (G.C.)

**Keywords:** nanoparticles, methylprednisolone, lung inflammation, COVID-19, ARDS

## Abstract

Background. Lipid–polymer hybrid nanoparticles (LPHNPs) offer a promising method for delivering methylprednisolone (MePD) to treat lung inflammation, addressing aggregation issues seen with polymer-only formulations. Objectives. This study aimed to develop LPHNPs for MePD delivery, assessing their physicochemical properties, drug loading, cytocompatibility, and release profiles, ultimately enabling inhalable microparticle-based powder. Methods. The nanoparticles were formulated using α,β-poly(N-2-hydroxyethyl)-DL-aspartamide-g-Rhodamine B-g-poly(lactic acid) (PHEA-*g*-RhB-*g*-PLA) and phospholipids DPPC, DOTAP, and DSPE-PEG2000 in a 45:30:25 weight ratio. Their size, redispersion after freeze-drying, drug loading (DL%), and controlled release were evaluated. Cytocompatibility was assessed on 16-HBE cell lines, measuring anti-inflammatory effects via IL-6 and IL-8 levels. Spray drying was optimized to produce microparticles using mannitol (MAN), leucine (LEU), and N-acetylcysteine (NAC). Results. The nanoparticles had a size of 186 nm and a DL% of 2.9% for MePD. They showed good cytocompatibility, significantly reducing IL-6 and IL-8 levels. Spray drying yielded microparticles with a fine particle fraction (FPF) of 62.3% and a mass median aerodynamic diameter (MMAD) of 3.9 µm. Inclusion of LPHNPs@MePD (0.25% *w*/*v*) resulted in FPF and MMAD values of 56.7% and 4.4 µm. In conclusion, this study described the production of novel inhalable powders as carriers for MePD-loaded nanostructures with favorable physicochemical properties, cytocompatibility, and promising aerosol performance, indicating their potential as an effective inhalable therapy for lung inflammation with corticosteroids, especially for treating chronic diseases.

## 1. Introduction

Systemic corticosteroid drugs are not only effective and widely available but also affordable, making them a standard treatment for several respiratory conditions, such as COVID-19 pneumonia, chronic obstructive pulmonary disease (COPD), asthma, and their exacerbations [1]. In the management of COVID-19, systemic corticosteroid therapy, predominantly using dexamethasone and methylprednisolone (MePD), has proven to facilitate the remission of inflammation, significantly reduce the risk of complications such as acute lung injury, and mitigate the development of acute respiratory distress syndrome (ARDS) [2,3]. These benefits extend even to high-risk patients, further demonstrating the critical role of corticosteroids in improving outcomes [4]. Research has shown that MePD can reduce mortality rates and shorten the duration of mechanical ventilation in patients suffering from ARDS [5,6]. MePD has been shown to be effective against the cytokine storm typical of lung inflammation [7]. Additionally, when combined with advanced antibiotics and antiviral drugs, methylprednisolone forms an effective regimen for treating viral pneumonia [8].

However, the chronic use of high-dose glucocorticoids is fraught with challenges. Numerous studies have highlighted that prolonged administration often results in hormone dependence and a range of severe side effects, including heightened susceptibility to double infections, diabetes, hypertension, osteonecrosis, and the suppression of the hypothalamic–pituitary–adrenal (HPA) axis, potentially leading to adrenal insufficiency [9]. Corticosteroids are also immunosuppressive, making patients more vulnerable to opportunistic infections [9].

In recent years, innovative approaches have emerged in the field of drug delivery aimed at addressing these challenges. For example, Raviv et al. introduced a novel lipid-based delivery system for systemic administration of MePD, specifically targeting lung tissue. This liposomal formulation demonstrated a significantly superior therapeutic effect compared to the free drug in treating ARDS, reducing inflammation, and decreasing TNFα, IL-6, and IL-1β cytokine levels in bronchoalveolar lavage fluid. Moreover, the study showed that combining intravenous (IV) and endotracheal (ET) nanoparticle delivery yielded the best outcomes [10].

In another paper, Su et al. reported the development of an inflammation-targeted biomimetic nanovehicle loaded with MePD, in which the cell membrane of mesenchymal stem cells was hybridized with liposomes [11]. Upon systemic administration, this nanovehicle exhibited remarkable targeting specificity toward inflamed lung cells, with minimal treatment-induced side effects.

However, chronic lung diseases requiring prolonged glucocorticoid therapy, systemic administration as well as oral administration, which is preferred due to better compliance, does not reduce the risk of serious side effects [1]. Consequently, the use of local delivery formulations is crucial to minimize systemic exposure and enhance drug accumulation in the target organ [10]. Inhaled corticosteroids have recently gained preference over oral or parenteral administration, as they require lower doses and deliver the drug directly to the lungs, thus minimizing systemic side effects [12,13].

Inhaled corticosteroids have long been used in combination therapies with β-agonists and muscarinic antagonists for managing diseases such as asthma and COPD [14,15]. These conventional formulations, often consisting of fine suspensions of pure drug particles, are associated with both local adverse effects and systemic effects.

The literature recently described innovative corticosteroid delivery systems for inhalation, leveraging strategies such as the use of polymeric nanoparticles [16,17].

In this context, Inapagolla et al. described the production of a MePD-polyamidoamine (PAMAM) dendrimer conjugate, which modifies the pharmacokinetic profile of the loaded drug upon trans-nasal method by retaining it in the lungs and providing controlled and sustained exposure compared to the inhalation of the drug alone [18,19]. Additionally, Liu et al. designed a biomimetic sustained release nanoplatform encapsulating MePD succinate, which has shown potential in treating acute lung inflammation by downregulating pro-inflammatory cytokines and promoting the production of anti-inflammatory cytokines in the lungs, after intravenous administration [20]. All these described systems, however, involve the use of colloidal dispersions intended for parenteral administration or aerosol delivery.

Recently, our research group has developed several inhalable formulations based on corticosteroid drugs for the treatment of inflammatory lung diseases [21,22], as well as hybrid systems for the delivery of other anti-inflammatory drugs, administered via inhalation [23,24].

The choice of hybrid systems is highly advantageous compared to the use of polymeric nanoparticulate systems or liposomes separately, as it combines the ability to incorporate large amounts of drugs and stability in biological fluids with the high biocompatibility of lipids [25,26]. Additionally, the presence of the shell can be suitably designed to modify the release profile of the drug embedded in the polymeric core.

Given the advantages associated with the use of microparticulate powders with suitable aerosolization properties and the need to optimize corticosteroid-based inhalation formulations until now described in the literature, in this work we describe the development of lipid–polymer hybrid carriers for the delivery of MePD locally to the lungs. To date, few studies report inhalable formulations containing MePD, the drug of choice for numerous inflammatory lung diseases currently administered intravenously. These formulations are made with biocompatible and functional excipients that can exert synergistic action to improve the characteristics of the mucus present in the airways. Additionally, the use of nanostructured systems allows for prolonged drug release compared to the drug’s dissolution, which can potentially reduce the required dosage. The components used to produce the hybrid particles were carefully selected to optimize the amount of loaded and released drug, to help overcome the physiological and anatomical barriers of the lung and enhance the interaction of the nanocarrier with lung cells. Furthermore, these hybrid systems were incorporated into microparticle matrices to enable administration via inhalation. The formulation of the microparticles was carefully optimized in terms of the qualitative and quantitative composition of the excipients and the aerodynamic properties, to allow inhalation administration and an active effect of the excipients on the characteristics of the treated area, with the aim of improving the performance of the hybrid nanoparticles and/or the loaded–released drug.

## 2. Materials and Methods

### 2.1. Materials

Dichloromethane (DCM), rhodamine B (RhB), anhydrous N,N′-dimethylformamide (DMF-a), 1,1′-carbonyldiimidazole (CDI), HPLC-grade acetonitrile (ACN), and deuterated N,N′-dimethylformamide (DMF-d7) were purchased from Sigma Aldrich (Milan, Italy), along with D,L-polylactic acid (PLA Acid Free, ${\bar{M}}_{w}$ = 10–18 kDa), L-leucine (LEU), N-acetylcysteine (NAC), D-mannitol, porcine stomach mucin, and methylprednisolone (MePD). Fluka (Milan, Italy) supplied diethylamine (DEA), acetone, and diethyl ether. Furthermore, 1,2-dipalmitoyl-sn-glycero-3-phosphocholine (DPPC), 1,2-dioleoyl-3-trimethylammonium-propane chloride salt (DOTAP), and 1,2-distearoyl-sn-glycero-3-phosphoethanolamine-N-(methoxy(polyethylene glycol)-2000) ammonium salt (DSPE-PEG2000) were obtained from Avanti Polar Lipids (Birmingham, AL, USA). Dialysis membranes were sourced from Spectrum Labs (Fisher Scientific Italia, Milan, Italy). All reagents used were of analytical grade.

Preparation and purification of α,β-poly(N-2-hydroxyethyl)-D,L-aspartamide (PHEA), PHEA-*g*-RhB, and PHEA-*g*-RhB-*g*-PLA followed previously established methods [27,28]. The average molecular weight ${\bar{(M}}_{w})$ of PHEA-*g*-RhB-*g*-PLA, as assessed via SEC analysis in an organic phase, was determined to be 86.9 kDa with akDa (${\bar{M}}_{w}/{\bar{M}}_{n}=$ 1.45).

### 2.2. Preparation of Empty and MePD-Loaded Polymeric Nanoparticles

Polymeric nanoparticles composed of PHEA-*g*-RhB-*g*-PLA, both unloaded (NPs) and loaded with MePD (NPs@MePD), were prepared via the dialysis method, following the approach of Cappelli et al. [29]. To prepare the nanoparticles, 90 mg of PHEA-*g*-RhB-*g*-PLA and 5 mg of MePD were first dissolved in 3 mL of DMSO. The resulting mixture was dialyzed for 24 h against double-distilled water using a Spectra Por membrane with a 100 kDa molecular weight cut-off (MWCO). Following dialysis, the nanoparticle dispersion was filtered through a 5 μm cellulose acetate membrane, rapidly frozen in liquid nitrogen, and subsequently freeze-dried with a ColdSafe PRO instrument, which operates at a temperature of −100 °C and a vacuum of 0.001 mBar. This process yielded NPs@MePD with a weight-based yield of 96%. Control nanoparticles (NPs) were prepared following the same procedure, but without the addition of MePD.

### 2.3. Preparation of Empty and Me-PD-Loaded Lipid−Polymer Hybrid Nanoparticles

Lipid−polymer hybrid nanoparticles were created using a two-phase process, beginning with the formation of lipid vesicles and followed by the integration of preformed polymeric nanoparticles. Initially, MePD-loaded liposomal vesicles were prepared via the Thin Layer Evaporation (TLE) technique as described by Craparo et al. [24]. Specifically, a mixture of 90 mg of DPPC, DOTAP, and DSPE-PEG_2000_ in a weight ratio of 45:30:25, along with 3 mg of MePD, was dissolved in 9 mL of chloroform (1% *w*/*v*). The solvent was then removed under reduced pressure with a rotary evaporator at 40 °C, resulting in a lipid layer, which was rehydrated with 5 mL of Milli-Q water and stirred at 75 °C for 30 min. Empty lipid vesicles were prepared using the same procedure without the addition of MePD.

In the second phase, lipid−polymer hybrid nanoparticles, both empty and MePD-loaded (referred to as LPHNPs and LPHNPs@MePD), were produced via high-pressure homogenization (HPH). The aqueous suspension of NPs or NPs@MePD was combined with the prepared lipid vesicles at a polymer-to-lipid weight ratio of 1:3. This mixture underwent six cycles of HPH at pressures between 10,000 and 15,000 psi, using an EmulsiFlex-C5 homogenizer. The resulting nanoparticle suspension was purified via ultracentrifugation at 40,000 RPM for 1 h at 25 °C. The supernatant was then removed, and the pellet was collected, freeze-dried, and stored for further analysis.

### 2.4. Characterization of Lipid−Polymer Hybrid Nanoparticles

#### 2.4.1. Dimensional Analysis and ζ Potential Measurement

The size distribution (in nm) and polydispersity index (PDI) of each sample were evaluated via Dynamic Light Scattering (DLS) [21]. Measurements were conducted on aqueous dispersions of each sample (0.2 mg/mL), both freshly prepared and post-lyophilization, using a Malvern Zetasizer NanoZS (Malvern Instruments, Worcestershire, UK) equipped with a 632.8 nm laser at a fixed scattering angle of 173°. The same instrument was employed to assess zeta potential, with values (mV) determined from electrophoretic mobility according to the Smoluchowski equation. All measurements were conducted in triplicate at 25 °C.

#### 2.4.2. Drug Loading (DL%) Determination

The amount of MePD incorporated into NPs@MePd and LPHNPs@MePD was evaluated via HPLC analysis, using an Agilent 1260 Infinity HPLC System equipped with a Luna 5u C_18_ column (Phenomenex, Bologna, Italy) and a mobile phase consisting of ACN/H_2_O (40:60 *v*/*v*), operating with a flow of 1 mL/min [30]. Briefly, the samples for HPLC analysis were prepared by dissolving a known amount of Nps@MePD and LPHNPs@MePD in 500 µL of DMSO, followed by the addition a known volume of mobile phase. After 1 h extraction under stirring, the dispersion was filtered through a regenerated cellulose (RC) filter of 0.22 μm and analyzed via HPLC. The analysis was performed in isocratic condition and the absorbance of MePD was detected at 238 nm (t_R_ = 6.8 min). Drug loading (DL%), defined as the percentage ratio of the weight of the encapsulated drug to the total weight of the sample, was determined by comparing the sample’s peak area against a calibration curve established from HPLC analysis of MePD standard solutions.

The drug calibration curve was obtained from seven different concentrations of MePD (0.0006, 0.0012, 0.003, 0.006, 0.012, 0.03, 0.12 mg/mL), by plotting the peak area of the drug against the nominal concentration of each standard solution which then were interpolated via linear regression (y = 212,118.3463x, R^2^ = 0.99985). Three calibration curves were prepared on three consecutive days, and each solution was injected three times. Sensitivity of the method was determined by means of the detection limit (LOD) and quantification limit (LOQ) [31]. The possible interference of the components of the hybrid nanoparticles was also evaluated via the same extraction procedure and HPLC method used for the LPHNPs@MePD, three different samples of empty LPHNPs alone or spiked with a solution of MePD (0.003 mg/mL).

#### 2.4.3. Release Study

The dialysis bag diffusion technique was employed to investigate the in vitro drug release of MePD from NPs@MePD and LPHNPs@MePD. For this purpose, 18 mL of three different simulated physiological fluids, phosphate buffer at pH 7.4, simulated lung fluid (SLF4), and artificial lysosomal fluid (ALF) [32], were added to three separate falcon tubes which represented the acceptor compartments. Dialysis tubing (12 kDa), containing 2 mL of NPs@MePD or LPHNPs@MePD dispersion (5 mg/mL), was immersed in each falcon tube, acting as the donor compartment. The system was maintained at 37 °C under continuous stirring at 100 rpm in an orbital shaker. At predetermined time intervals, aliquots of 1 mL were collected from the acceptor compartment and replaced with an equal volume of fresh medium to maintain the sink conditions. Each sample was subsequently lyophilized, and the resulting solid was analyzed via HPLC, following the procedure described above. Each experiment was performed in triplicate.

#### 2.4.4. Cell Viability

The cytocompatibility of LPHNPs and LPHNPs@MePD was assessed using an MTS assay on human bronchial epithelium (16-HBE) cells, with the commercially available kit Cell Titer 96 Aqueous One Solution Cell Proliferation assay (Promega, Milan, Italy). Cells were seeded into a 96-well plate at a density of 2.5 × 10^4^ cells per well and cultured in DMEM supplemented with 10% fetal bovine serum and 1% penicillin/streptomycin (10,000 U/mL penicillin and 10 mg/mL streptomycin) at 37 °C in a 5% CO₂ atmosphere. After 24 h, the culture medium was exchanged with 200 μL of fresh medium containing LPHNPs or LPHNPs@MePD at various concentrations, corresponding to MePD equivalent concentrations of 0.75, 1.5, 3.75, 7.5, and 15 μg/mL [33]. Free MePD dispersions at matching concentrations served as positive controls, while untreated cells acted as negative controls. Following 24 and 48 h of incubation, the medium was replaced with 100 μL of fresh culture medium along with 20 μL of MTS reagent. The cells were then incubated for an additional 2 h at 37 °C, after which absorbance was measured at 492 nm using a microplate reader (PlateReader AF2200, Eppendorf, Hamburg, Germany). Each experiment was conducted in triplicate, with cell viability expressed as a percentage, calculated by comparing each sample’s absorbance to that of the negative control (representing 100% viability).

#### 2.4.5. ELISA Test

The assessment of the anti-inflammatory effects of MePD encapsulated within hybrid systems, was performed via ELISA assays, which were conducted utilizing the IL-6 Human ELISA Kit from Cayman Chemical and the IL-8 Human ELISA Kit from Sigma Aldrich [33]. Briefly, 16-HBE cells were plated in a 96-well plate at a density of 2.5 × 10^4^ cells per well in DMEM and incubated for 24 h. Subsequently, the cells were activated with LPS (10 µg/mL) and simultaneously treated with MePD (7.5 µg/mL), LPHNPs, and LPHNPs@MePD (0.25 mg/mL). After an additional 24 h incubation, supernatants were collected to measure IL-6 and IL-8 levels using the respective ELISA kits, following the manufacturer’s instructions. LPS-stimulated cells and unstimulated cells treated with DMEM served as controls. Each formulation was tested in triplicate, and results were reported as mean ± SD.

### 2.5. Microparticle Production and Characterization

#### 2.5.1. Preparation of Spray-Dried Microparticles (MPs) and Nano into Microparticles (NiM)

To produce spray-dried microparticles (MPs), dispersions in H_2_O: EtOH (95:5) mixture containing D-mannitol (MAN) (6, 8, 10, or 12% *w*/*v*), leucine (LEU) (2% *w*/*v*) and N-acetylcysteine (NAC) (2% *w*/*v*) were prepared.

The MPs production was carried out using a Mini Spray Dryer B290 (Buchi, Milan, Italy) under the following conditions: inlet temperature was set to 110 °C, while the outlet temperature ranged from 60 to 66 °C [34]. Aspiration rates were maintained at either 100% or 70%, and the feed pump operated at 10% or 7%. The atomizer nozzle had a diameter of 140 µm, and compressed air was utilized as the gas source. The experimental parameters, along with the qualitative and quantitative composition of the liquid feed, are detailed in Table 1. Nano into Microparticles formulation (referred to as NiM@MePD) were produced by using dispersions in the H_2_O: EtOH (95:5) mixture containing MAN (10% *w*/*v*), LEU (2% *w*/*v*), NAC (2% *w*/*v*), and LPHNPs@MePD (0.25% *w*/*v*) as a feeding liquid, and by setting the feed pump at 7% and the aspiration at 100% (see Table 1).

#### 2.5.2. Particle Morphology

The morphology of different MPs and NiM@MePD obtained was evaluated using scanning electron microscopy (SEM) (Phenom™ ProX Desktop SEM microscope, Thermo Fisher Scientific, Milan, Italy) [23]. The sample was affixed to double-sided adhesive tape that had been previously applied to a stainless-steel stub and then sputter-coated with a thin layer of gold (approximately 10 nm) prior to microscopy analysis. The acceleration voltage was 10–15 kV. Particle size distribution and geometric diameter (d_geom_) for each sample were assessed by analyzing more than 500 particles using ImageJ software 1.8.0.

#### 2.5.3. Determination of Tapped Density (ρ_tapped_), Aerodynamic Diameter (d_aer_), and Hausner Index (H)

The *ρ*_tapped_ of MPs samples and NiM@MePD was measured using the syringe method [35]. Each powder sample was filled into a 1 mL graduated syringe to measure the uncompacted powder volume (bulk volume). The tapped volume was then determined by tapping the syringe on a flat surface 100 times until the volume remained constant. Bulk and tapped densities (ρ_bulk_ and ρ_tapped_, respectively) were then calculated by dividing the weight of the powder by the respective volumes. All measurements were conducted in triplicate [23].

The theoretical aerodynamic diameter (d_aer_) was calculated using the following equation, valid for particles with a diameter greater than 0.5 μm:

(1) $$d_{aer}=d_{g}\sqrt{\rho \;\chi \cdot \rho 0}$$

where:

d_g_ is geometric diameter (μm)

*ρ* is tapped density (g/cm^3^)

χ is shape factor (equal to 1 for spherical particles)

*ρ*_0_ is unit density

The Hausner Index (H) was calculated from the bulk and tapped density values to evaluate the flowability of the powders using the following formula:

(2) $$H=(\rho_{tapped}/\rho_{bulk})$$

where:

*ρ*_bulk_ is bulk density (g/cm^3^)

*ρ*_tapped_ is tapped density (g/cm^3^)

#### 2.5.4. Measurement of Interactions with Mucin

The interactions between MPs_C1, NiM@MePD and LPHNP@MePD samples with mucin were studied via turbidimetric analysis [36]. Briefly, Type II mucin dispersion was prepared by dispersing an excess amount of mucin in water (0.16% *w*/*v*), stirring it overnight. The dispersion was then centrifugated at 6000 rpm at 4 °C for 20 min and the supernatant was collected and used for the experiment. Each microparticle sample was dispersed in water at a concentration of 2 mg/mL, mixed 1:1 (*v*/*v*) with the mucin dispersion, and the resulting mixture incubated in an orbital shaker at 37 °C, for 30 min and 1 h. At predetermined time intervals, light scattering measurements of the mucin–sample aqueous mixtures were conducted using spectrophotometric analysis at a wavelength of 650 nm. Additionally, the absorbance of each aqueous dispersion containing either mucin or individual samples was assessed as a control. All experiments were carried out in triplicate and the results are presented as absorbance at 650 nm ± SD over the duration of the study.

#### 2.5.5. Andersen Cascade Impactor (ACI) Analysis

The aerosolization characteristics of the spray-dried formulations were evaluated using an Andersen cascade impactor (ACI) device (InPharmaTEC, Cogliate (MB), Italy). An inhalation flow rate of 90 L/min was maintained with a pump (Bavo X BIO, TCR TECORA^®^, Cogliate, Italy) [37]. The cut-off aerodynamic diameters for the stages of the ACI at this flow rate are detailed in Table 2. All formulations were aerosolized using a Breezhaler^®^ single-dose device (Novartis International AG, Basel, Switzerland), utilizing size 3 gelatin capsules filled with 45 mg of MPs or NiM@MePD samples [37]. The liquid feed for these formulations included Rhodamine at a concentration of 0.05% *w*/*v*, as described in Table 1.

To ensure optimal particle deposition during powder aerosolization, the impaction cups of each stage were pre-coated with a 1% (*w*/*v*) solution of Tween 80^®^ in ethanol and allowed to dry for 30 min prior to use. Five manually filled capsules were then sequentially introduced into the ACI, with each capsule discharging for 5 s. Afterward, the deposited powder from each stage was collected using 2 mL of water, and absorbance intensities were measured at a wavelength of 540 nm. Standard curves were generated using dispersions of MPs and NiM@MePD at varying concentrations.

For each sample, the experiment was conducted a minimum of three times, and the resulting data were analyzed to determine the emitted powder (EP: amount of powder leaving the devise and reaching the impactor), the EP% (ratio between the EP and the initial mass of the tested sample), and the IP% (ratio between the inhaled powder and the EP). Additionally, the fine particle fraction (FPF%) was calculated as the percentage of EP with an aerodynamic diameter of less than 5.0 µm, obtained by interpolating the cumulative aerodynamic diameter distribution curve. The mass median aerodynamic diameter (MMAD) and the geometric standard deviation (GSD) of the particles’ aerodynamic diameter were derived from the graph, following the guidelines of the *European Pharmacopeia* (11th edition).

## 3. Results and Discussion

**Scheme 1:**
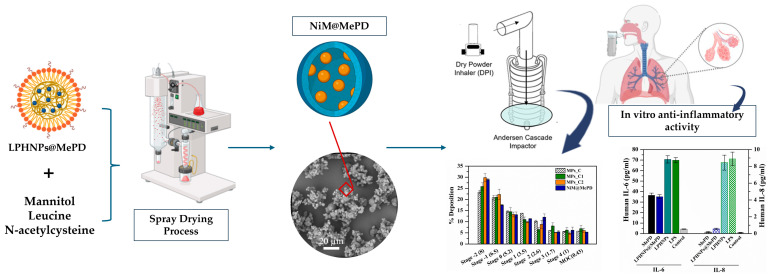
Schematic representation of the Nano into Microparticle (NiM) production starting from hybrid nanoparticles (LPHNPs@MePD) and excipients (Mann, LEU, NAC), and characterization.

In this work, we focused on the design, development, and characterization of an innovative inhalable formulation, using the Nano into Micro (NiM) approach through the spray drying (SD) technique, for the administration of methylprednisolone (MePD) in the treatment of lung inflammation [1,4]. In Scheme 1, the aim of the work is graphically summarized.

### 3.1. Preparation and Characterization of Empty and MePD-Loaded Lipid–Polymer Hybrid Nanoparticles (LPHNPs)

The copolymer α,β-poly(N-2-hydroxyethyl)-DL-aspartamide-*g*-RhodamineB-*g*-poly(lactic acid) (PHEA-*g*-RhB-*g*-PLA), used for obtaining the polymeric core of the hybrid particles, was synthesized as reported elsewhere [22,27]. In particular, empty or MePD-loaded polymeric nanoparticles (NPs) were obtained by using the dialysis method starting from a PHEA-*g*-RhB-*g*-PLA dispersion of in dimethyl sulfoxide (DMSO) in the presence or not in the presence of the drug. The amount of MePD incorporated into polymeric nanoparticles, expressed as drug loading (DL%), was found to be 1.2% *w*/*w*.

**Figure 1:**
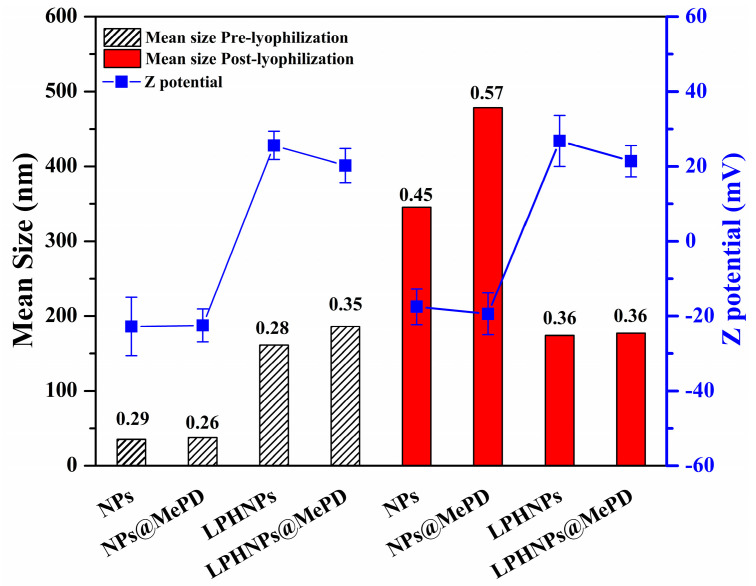
Z Average, PDI, and ζ potential values of empty and drug-loaded NPs and LPHNPs, before and after lyophilization. The number above the histogram represents the PDI of the Z average.

The obtained empty or drug-loaded nanoparticle samples (named NPs and NPs@MePD, respectively), were characterized by using DLS analysis to determine the mean diameter (expressed as Z average), the polydispersity index (PDI), and the zeta potential. The characterization was repeated after the dried process, to evaluate their redispersibility. The obtained values, before and after freeze-drying, are reported in Figure 1.

As evident from the reported data, both samples had a comparable mean size, and showed a low polydispersity. In both cases, the ζ potential was negative and unaffected by the presence of the drug. After freeze-drying however, neither sample easily redispersed, making the samples unsuitable for direct use in the production of a formulation.

For this reason, it was decided to optimize the formulation using phospholipids. In particular, liposomal vesicles, which are necessary to realize LPHNPs, were obtained via Thin Layer Evaporation (TLE) from a mixture of three phospholipids: 1,2-dipalmitoyl-sn-glycero-3-phosphocholine (DPPC), 1,2-dioleoyl-3-trimethylammonium propane chloride (DOTAP), and 1,2-distearoyl-sn-glycero-3-phosphoethanolamine-N-(methoxy(polyethylene glycol)-2000) (DSPE-PEG_2000_) (in a weight ratio of 45:30:25), and MePD in chloroform (0.03% *w*/*v*).

DPPC was chosen as the main endogenous constituent of pulmonary surfactant and, therefore, biocompatible and well-tolerated. Due to its zwitterionic nature, it ensures an adequate zeta potential that stabilizes the LPHNPs, preventing their aggregation [22]. DOTAP was selected because it has a permanent positive charge and can modulate the charge of resulting nanostructured systems, thereby increasing stability. DSPE-PEG_2000_ was chosen to obtain surface-pegylated nanostructures, characterized by easy redispersion in aqueous media and reduced interaction with mucins in mucus.

LPHNPs were prepared from a dispersion of polymeric nanoparticles and liposomal vesicles using the high-pressure homogenization (HPH) method. As for the NPs, the obtained LPHNPs, both before and after lyophilization, were analyzed via DLS to obtain information on the Z average, PDI, and ζ potential; data are reported in Figure 1.

Both empty and drug-loaded hybrid nanoparticles (named LPHNPs and LPHNPs@MePD) exhibited a comparable mean size, ranging between 161 and 186 nm, and good polydispersity, which showed a slight increase in the presence of the drug. After the lyophilization process, there were no significant variations in either size or polydispersity after redispersion in distilled water of hybrid samples. This result could be explained by the presence of the phospholipid shell on the obtained particles, which limits their aggregation and facilitates redispersion in the aqueous medium. The ζ potential values of the particles were positive and not significantly influenced by either of the drug presence or the lyophilization process and redispersion in aqueous medium.

In the literature, red blood cells (RBC), hitchhiking chitosan nanoparticles loading methylprednisolone, are described for the intravenous route. These nanoparticles showed a more comparable mean size and zeta potential values, as well as drug release profile, than our hybrid systems. Moreover, these showed in vivo preferential accumulation into the lung, inducing a significant reduction in cytokine storm thanks to the adsorption on the RBC surface, while free nanoparticles were cleared by the RES [7].

Other authors have recently described liposomal systems as carriers for the intravenous delivery of methylprednisolone succinate, which exhibited sizes around 100 nm, a negative zeta potential, and a slow release in 5% dextrose solution [10]. The choice of carrier type is clearly dictated by the form in which the drug is present, as methylprednisolone succinate is highly soluble in water. Other authors report the preparation and characterization of liposomes containing methylprednisolone targeted to the lungs through the use of a humanized surfactant protein-A nanobody, which exhibited small sizes and showed encouraging in vivo results in animals following intratracheal injection [38,39]. Few systems, however, are currently documented in the literature for methylprednisolone aimed at local administration and targeted action in the lungs. Certainly, there is little focus on the type of administration and on formulations specifically designed for dry powder inhalation using systems already in clinical use.

The DL%, which in this case represents the MePD amount incorporated into the hybrid system, was found to be 2.9% *w*/*w*. The increase in DL% value observed in the hybrid nanoparticles compared to the polymeric nanoparticles (DL% = 1.2% *w*/*w*) confirms that an additional amount of the drug is incorporated during the lipid vesicle formation phase. This suggests that the lipid shell plays a significant role in enhancing the drug encapsulation efficiency, likely due to its ability to interact with and retain the drug molecules within the vesicular structure.

### 3.2. Release Study

To evaluate the release profile of MePD loaded into the LPHNPs@MePD sample, a cumulative release study was carried out in various fluids simulating different interstitial environments, such as extracellular fluid (DPBS at pH 7.4), simulated pulmonary alveolar fluid (SLF4) containing 0.02% *w*/*v* DPPC, and artificial lysosomal fluid (ALF) at pH 4.5, to mimic the fluid with which inhaled particles come into contact after potential phagocytosis by alveolar and interstitial macrophages [32].

**Figure 2:**
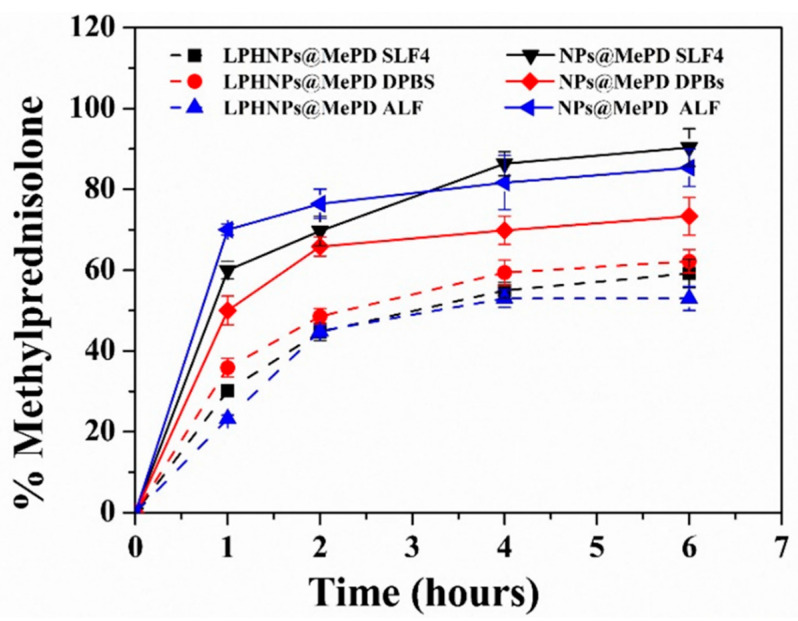
MePD release profile from LPHNPs (dashed line) and NPs@MePD (solid line), in SLF4 (black), DPBS (red), and ALF (blue).

The obtained release profiles (Figure 2), reported as the % of drug amount released on the total amount contained in the samples as a function of incubation time, show that the loaded drug into hybrid nanoparticles is slowly released, up to 51%, 57%, and 47% *w*/*w* in SLF4, DPBS, and ALF, respectively, after 6 h. Notably, the release profiles were similar across different incubation media, indicating that the release mechanism is independent of the fluid environment.

For comparison, the drug release from polymeric nanoparticles (NPs@MePD) was also evaluated. Compared to the LPHNPs@MePD, the polymeric nanoparticles exhibited a higher drug release rate across all media. Moreover, like for the hybrid system, the release was not significantly influenced by the incubation conditions. This comparison underscores the role of the lipid shell in the hybrid nanoparticles, which modulates the release profile by slowing the drug release. The lipid layer effectively acts as a barrier, resulting in a more controlled and sustained release compared to the faster release observed in polymeric nanoparticles. This highlights the advantage of the hybrid system in providing a more gradual drug release, which may be beneficial for maintaining therapeutic levels over extended periods.

### 3.3. Biological Assays

**Figure 3:**
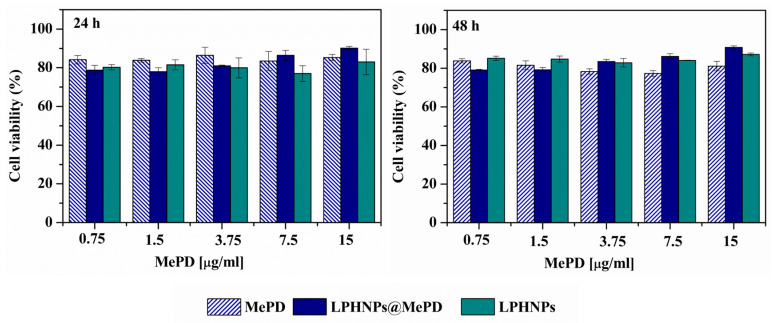
Cell viability % (MTS assay) on 16-HBE cells after 24 and 48 h of incubation with MePD, LPHNPs@MePD, and LPHNPs. The results are reported as the mean ± SD (n = 3).

To evaluate the potential use of the hybrid nanoparticles as therapeutic tools for treating lung inflammation, their cytocompatibility was evaluated via the MTS assay on 16-HBE cells as a function of hybrid nanoparticles concentration (LPHNPs and LPHNPs@MePD) after 24 and 48 h of incubation (Figure 3). MePD concentrations were chosen in a range according to other studies [33].

As can be seen, after 24 and 48 h of incubation, both LPHNPs and LPHNPs@MePD showed an excellent cytocompatibility at all tested concentrations, as cell viability was higher than 85% compared to the control.

Once it was demonstrated that the samples were not toxic to 16-HBE epithelial cells, the anti-inflammatory efficacy of MePD loaded into LPHNPs was evaluated by quantifying IL-6 and IL-8 levels produced by 16-HBE cells following stimulation with LPS [33,40]. This approach provides a well-established model to explore the anti-inflammatory potential of therapeutic agents, as IL-6 and IL-8 are key pro-inflammatory cytokines that play a central role in inflammatory responses, especially within the context of lung inflammation.

**Figure 4:**
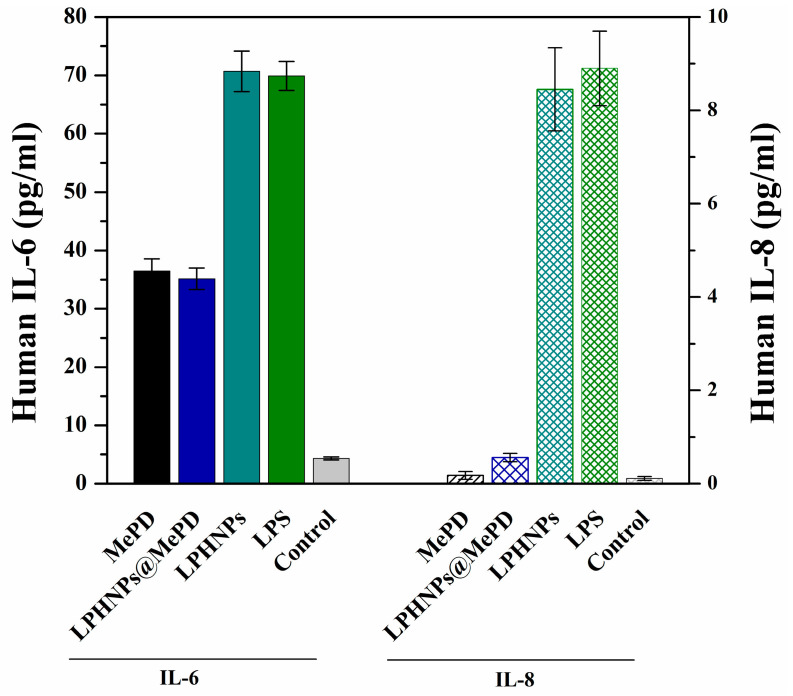
Evaluation of MePD, free or loaded into LPHNPs, and empty LPHNPs effects on 16-HBE cells as cytokine IL-6 e IL-8 production via ELISA test.

The results obtained via ELISA test (Figure 4) showed that LPS stimulation significantly increased the production of both IL-6 and IL-8, which are known to mediate and amplify the inflammatory response. Notably, when cells were co-incubated with LPS and MePD-loaded hybrid nanoparticles, a marked reduction in IL-6 and IL-8 levels was observed. This reduction is comparable to the levels observed with an equivalent concentration of MePD, indicating that the LPHNPs@MePD effectively delivered the drug to the cells and facilitated its anti-inflammatory action.

Therefore, the produced LPHNPs systems are potentially ideal carriers for MePD, as they provide a slow and sustained release, unaffected by the incubation medium. Furthermore, unlike polymeric nanoparticles, which often require high amounts of cryoprotectants to prevent aggregation during freeze-drying, LPHNPs can be freeze-dried and redispersed in aqueous solutions without incurring aggregation phenomena. This property simplifies their handling and storage, making them more practical for possible clinical use.

### 3.4. Microparticle Preparation and Characterization

Due to their colloidal size, these nanoparticles cannot be administered in the form of dry powder, as they would be lost during exhalation. However, they can be effectively delivered to the lungs via nebulization after dispersion in a physiological medium or incorporated into inhalable powders; nebulization is a process that requires time and the use of devices whose types and modes of operation increase the variables that can affect the proper administration of the formulation, in addition to those closely related to the pathology, the characteristics of the site of administration, and the patient.

To enable this, the Nano into Micro (NiM) strategy was employed, where microparticles containing LPHNPs@MePD were produced via the spray drying (SD) technique and using biocompatible hydrophilic excipients to form the micrometric matrix. We suppose that, once inhaled, the hydrophilic matrix of the microparticles will rapidly dissolve and release the incorporated colloidal particles. These nanoparticles, with their optimized size and surface properties, can then diffuse through the mucus and reach the lung epithelium, facilitating effective drug delivery to the target site.

Here, excipients with well-established roles in enhancing the production of inhalable powders were strategically selected for the matrix formulation. These include D-mannitol (MAN), L-leucine (LEU), and N-acetylcysteine (NAC). Mann, as an osmotic agent, is able to improve the hydration of lung mucus, increasing its fluidity; NAC has a mucolytic action due to its thiol groups [10,41]. Additionally, it has been observed that its use can potentially hinder the binding of the SARS-CoV-2 Spike Protein to the ACE-2 receptor, a key factor in the development of acute respiratory distress syndrome (ARDS). It also neutralizes oxidized compounds generated in lung fluids during infections, contributing to minimizing tissue damage caused by oxidative stress.

However, despite these beneficial properties, NAC has the tendency to reduce flowability and increase adhesiveness of the final powder. This limitation has been overcome by adding LEU, a widely used excipient in dry powder formulations. It not only improves powder dispersibility and aerosolization, thereby enhancing the emitted dose and fine particle fraction, but also plays a crucial role in stabilizing particle physicochemical properties. Specifically, it prevents degradation due to moisture, a common challenge in dry powder formulations [42].

**Figure 5:**
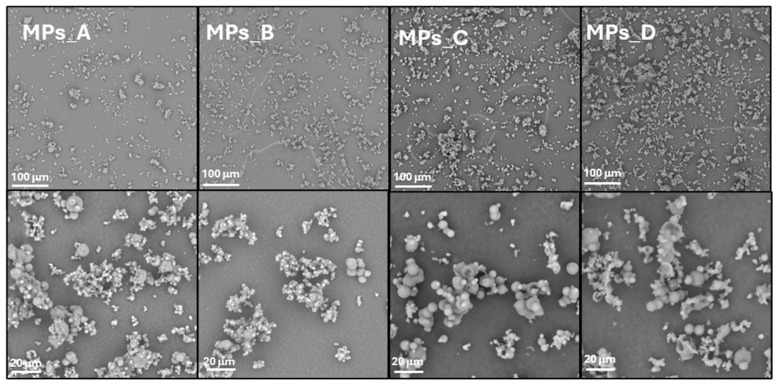
SEM images depicting samples MPs_A, MPs_B, MPs_C, and MPs_D with magnifications of 500× (upper) and 3000× (down).

Using a Buchi B290 spray dryer, a series of microparticle samples were produced using dispersions of excipients only in the absence of LPHNPs to optimize the microparticle production process and find suitable operating conditions for the realization of NiM. In particular, starting from ethanolic–aqueous dispersions (H_2_O:EtOH 95:5 *v*/*v*) at constant concentration of LEU and NAC (2% *w*/*v*), empty microparticle matrices were obtained at MAN concentrations equal to 6, 8, 10, and 12% *w*/*w*, named respectively MPs_A, MPs_B, MPs_C, and MPs_D. Moreover, the process parameters such as pump and aspirator were kept constant (see Table 1 in the Section 2). SEM images acquired on obtained samples are shown in Figure 5.

**Figure 6:**
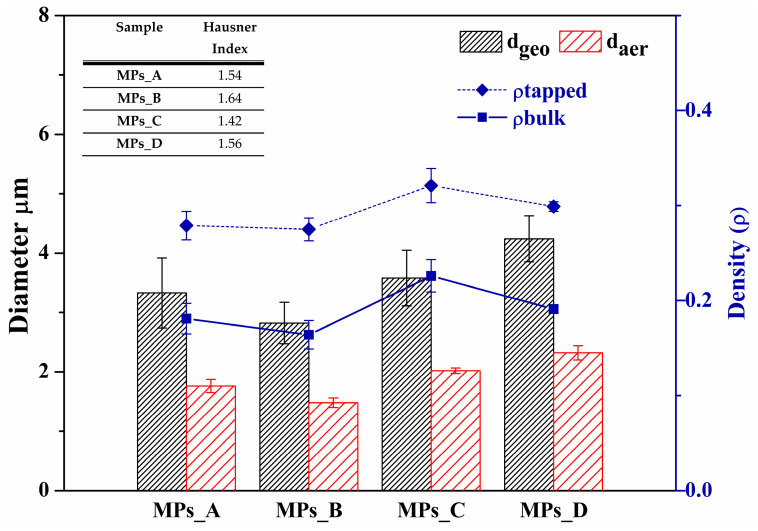
Characteristics of the samples obtained via SD in terms of geometric diameter (d_geo_), bulk density (ρ_bulk_), tapped density (ρ_tapped_), aerodynamic diameter (d_aer_), and Hausner Index (H).

The determination of the geometric diameter (d_geo_) was conducted via dimensional analysis on the SEM images, while the tapped density (ρ_tapp_) measurement was performed on the powders, from which the Hausner Index was calculated [35]. From Equation (1) of the Section 2, the theoretical aerodynamic diameter (d_aer_) was calculated. All the data obtained are presented in Figure 6.

From the analysis of these data, it can be concluded that all the samples showed values of d_aer_ falling within the acceptable range for efficient aerosolization (1–5 μm); MPs_B showed the lowest value of d_aer_ but the highest flowability index compared to the others. Therefore, considering all parameters and morphology, the sample MPs_C was selected. This sample exhibited spherical particles, with adequate values in terms of size (d_geom_ and d_aer_ equal to 3.58 and 2.02 μm, respectively) and a lower Hausner Index (H = 1.42) compared to the others, indicating better powder flowability. Moreover, as clearly seen in the SEM images, the sample exhibited single particle diameter comparable to the mean size, while MPs_B consisted of very small particles aggregated together (which justified its lower flowability tendency).

**Figure 7:**
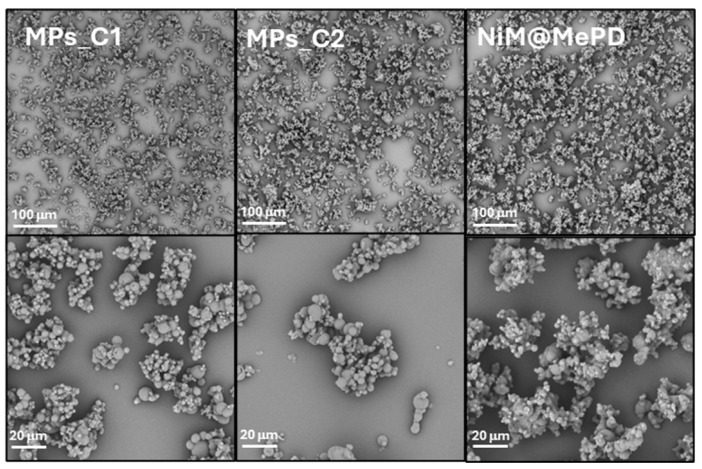
SEM images depicting samples MPs_C1, MPs_C2, and NiM@MePD (containing LPHNPs at 0.25% *w*/*v*), with magnifications of 500× (upper) and 3000× (down).

**Figure 8:**
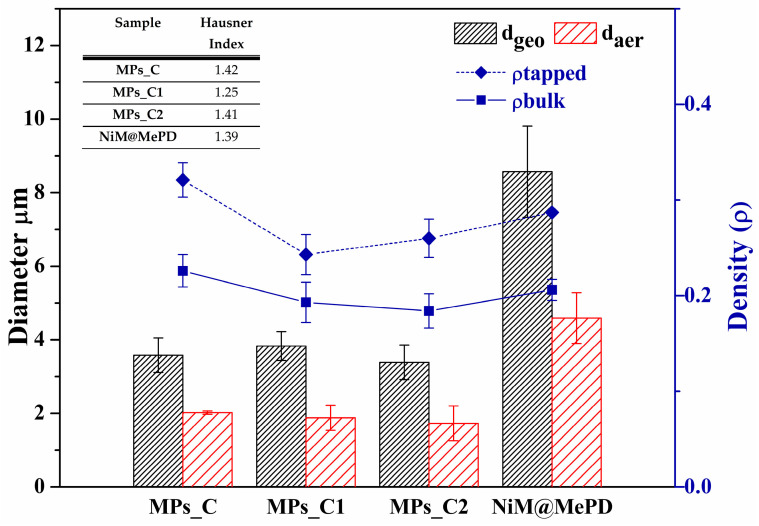
Geometric diameter (d_geo_), bulk density (ρb), tapped density (ρ_tapped_), aerodynamic diameter (d_aer_), and Hausner Index (H) of MPs_C1, MPs_C2 and NiM@MePD samples. MPs_C sample is reported for comparison.

Before proceeding with the preparation of the NiM, two other microparticle samples were produced. The qualitative and quantitative composition of the liquid feed was kept the same as that used for the MPs_C sample, while the pump and aspirator settings were varied, as detailed in Table 1 (see the Section 2). The SEM images of the obtained samples, named MPs_C1 and MPs_C2, and the results of the technological characterization are shown in Figure 7 and Figure 8, respectively.

In terms of morphology, the produced particles MPs_C1 and MPs_C2 were more spherical and homogeneous compared to the MPs_C sample. Moreover, values of d_aer_ and ρ_tapped_ were almost comparable to those of the MPs_C sample. All these characteristics positively influenced flowability, which was optimal for the MPs_C1 sample (H = 1.25).

To obtain additional information on aerosolization properties, the evaluation of the aerosolization performance was performed on samples MPs_C, MPs_C1, and MPs_C2 by using the Andersen cascade impactor (ACI) device. This method provides critical information on particle size distribution and deposition patterns, which are essential for assessing the efficacy of inhalable formulations.

**Figure 9:**
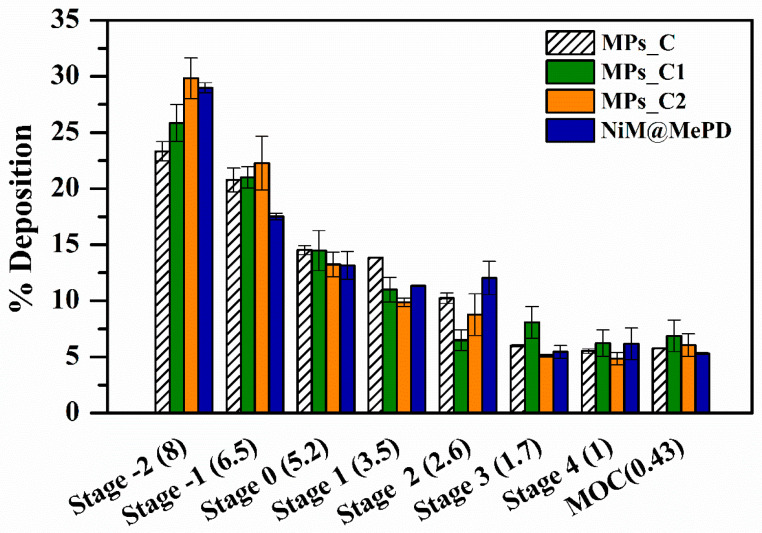
Deposition of MPs_C, MPs_C1, MPs_C2 and NiM@MePD samples on the stages of the ACI, after testing via Breezhaler^®^ at a flow rate of 90 L/min.

The results, presented in Table 3 and Figure 9, offer a comparative analysis of the three samples, highlighting any variations in fine particle fraction (FPF) and emitted powder (EP%).

All parameters indicated that the MPs_C1 sample was the most suitable for optimal deposition after aerosolization, showing FPF of 62.3% and MADD equal to 3.9 μm.

### 3.5. Nano into Micro (NiM) Production and Characterization

Therefore, the composition of the feed fluid (10% *w*/*v* Mann, 2% *w*/*v* LEU, 2% *w*/*v* NAC) as well as the SD parameters used to produce this sample (7% pump, 100% aspiration) were chosen to produce microparticles in which to incorporate the hybrid nanoparticles.

In particular, to produce NiM (named NiM@MePD), the LPHNPs@MePD sample was added at 0.25% *w*/*v*. The SEM images of the obtained sample are shown in Figure 7, while the values of d_geo_, ρb, ρ_tapped_, d_aer_, and H are reported in Figure 8.

As observed, the addition of LPHNPs to the feeding fluid of the SD process increased the heterogeneity of the microparticles, whose higher presence of particulate aggregates compared to the MPs_C1 sample led to an increase in the values of d_geo_, ρ_bulk_, ρ_tapped_, d_aer_, and H. Moreover, the MMAD value of NiM@MePD was also higher in respect to the MPs_C1 sample (4.4 vs. 3.9 µm), although falling within the range for efficient aerosolization in the lower airways. Therefore, the optimization of the matrix production process represents an excellent starting point for the preparation of NiM containing nanoparticulate systems for the delivery of MePD to the lungs.

**Figure 10:**
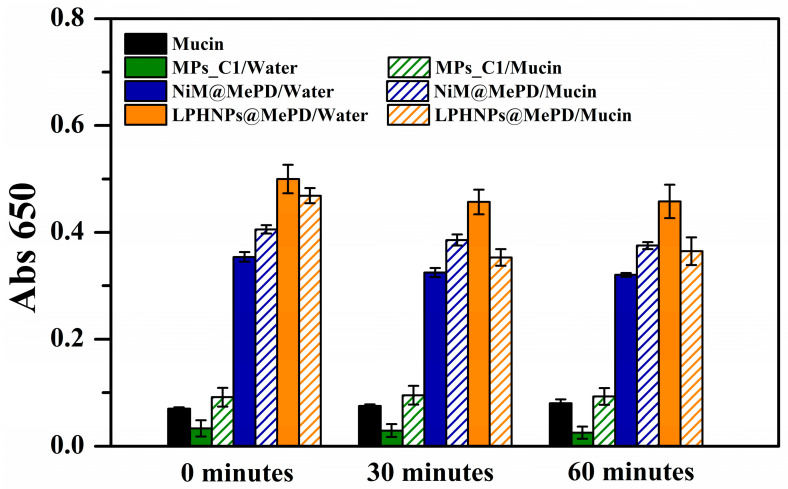
Transmittance at 650 nm of dispersions containing mucin alone, MPs_C1, NiM@MePD and LPHNPs@MePD samples (1 mg/mL), in water or in mucin dispersion at final concentration of 0.08% *w*/*v*.

To assess the tendency of these particles to interact with pulmonary mucus, a turbidimetric test was conducted in which the extent of interactions between NiM@MePD and mucin was measured. Results were compared with results obtained with the microparticle sample in the absence of hybrid particles (MPs_C1 sample) and the hybrid particles alone (LPHNPs@MePD). The analysis was also conducted in the absence of mucin, and results are reported in Figure 10.

A significant increase in the absorbance at 650 nm in the dispersion of NiM@MePD sample with mucin was observed when compared to that in the absence of mucin (*p* < 0.005). This result suggests that the system had a certain capacity for interaction with mucin under the chosen experimental conditions. This effect was also observed for the MPs_C1 sample, suggesting that components of the microparticle matrix, including N-acetylcysteine (NAC), may play a key role in facilitating interaction with mucin. NAC’s known mucolytic properties, along with its thiol groups, could enhance binding to mucin by disrupting mucus structure or through direct chemical interaction, further supporting the observed results [41].

On the other hand, LPHNPs did not show a significant change in absorbance in the presence of mucin, suggesting that they did not interact with the latter. Therefore, LPHNPs, once released from the microparticle matrix, may retain the ability to diffuse freely through the mucus layer without interactions with mucin chains. Such behavior is advantageous for drug delivery systems aimed at penetrating the mucus barrier and delivering therapeutic agents to underlying tissues.

## 4. Conclusions

In conclusion, this study was focused on the realization of an inhalable powder formulation of methylprednisolone (MePD) aimed at treating pulmonary inflammatory conditions, including COVID-19-associated ARDS, with reduced systemic side effects. By encapsulating MePD in lipid–polymer hybrid nanoparticles (LPHNPs) and embedding these into spray-dried microparticulate matrices, optimized to contain mannitol, leucine, and N-acetylcysteine (NAC), the formulation enables targeted pulmonary delivery with controlled release. The microparticles, optimized for aerosolization, demonstrate suitable aerodynamic properties for deep lung deposition and efficient mucus diffusion. Turbidimetric studies revealed minimal interaction with mucin, supporting nanoparticle diffusion through lung mucus post-matrix dissolution, while NAC in the matrix may aid in mucus network disassembly, further enhancing mucus penetration. The formulation’s hybrid nanoparticles were designed for cytocompatibility and effective anti-inflammatory action, significantly reducing IL-6 and IL-8 levels, confirming its therapeutic potential in lung inflammation. Overall, the NiM-based approach combines local, controlled drug release, biocompatibility, and minimal mucus interaction, offering a promising therapeutic strategy for managing ARDS and similar conditions while minimizing systemic side effects.

## Figures and Tables

**Table 1:** Composition of the samples and experimental parameters used to produce the microparticle matrix and NiM (with a 140 μm nozzle and a 110 °C T inlet).

Sample	Mann (wt/v%)	LEU (wt/v%)	NAC (wt/v%)	Pump (%)	Aspirator (%)
MPs_A	6	2	2	10	100
MPs_B	8	2	2	10	100
MPs_C	10	2	2	10	100
MPs_D	12	2	2	10	100
MPs_C1	10	2	2	7	100
MPs_C2	10	2	2	10	70
NiM@MePD	10	2	2	7	100

**Table 2:** Cut-off aerodynamic diameter for stages of Andersen cascade impactor (ACI) at a flow rate of 90 L/min.

ACI Stages	Cut-Off Diameter at 90L/min (µm)
−2	8
−1	6.5
0	5.2
1	3.5
2	2.6
3	1.7
4	1
MOC	0.43

**Table 3:** Aerodynamic behavior of samples, expressed as emitted powder (EP%), inhaled powder (IP%) respect the emitted powder, fine particles fraction (FPF), mass medium aerodynamic diameter (MMAD), and geometric standard deviation (GSD).

Sample	EP%	IP%	FPF%	MMAD (μm)	GSD (μm)
MPs_C	48.6 ± 1.7	56.4 ± 1.7	58.3 ± 1.6	4.3 ± 0.6	1.6 ± 0.3
MPs_C1	57.8 ± 2.5	38.9 ± 1.6	62.3 ± 0.3	3.9 ± 0.1	1.8 ± 0.1
MPs_C2	42.6 ± 2.5	51.4 ± 3.2	52.7 ± 2.7	4.7 ± 0.3	1.5 ± 0.2
NiM@MePD	34.2 ± 0.7	41.9 ± 2.2	56.7 ± 0.1	4.4 ± 0.1	1.6 ± 0.1

## Data Availability

Data are contained within the article.

## List of Abbreviations

1,1′-carbonyldiimidazole (CDI);1,2-dioleoyl-3-trimethylammonium-propane chloride salt (DOTAP); 1,2-dipalmitoyl-sn-glycero-3-phosphocholine (DPPC); 1,2-distearoyl-sn-glycero-3-phosphoethanolamine-N-(methoxy(polyethylene glycol)-2000) ammonium salt (DSPE-PEG2000); acetonitrile (ACN); acute respiratory distress syndrome (ARDS); aerodynamic diameter (d_aer_); anhydrous N,N′-dimethylformamide (DMF-a); artificial lysosomal fluid (ALF); bulk density (r_bulk_); chronic obstructive pulmonary disease (COPD); D,L-polylactic acid (PLA); deuterated N,N′-dimethylformamide (DMF-d7); dichloromethane (DCM); diethylamine (DEA); D-mannitol (MAN); drug loading (DL%); Dynamic Light Scattering (DLS); emitted powder (EP%); endotracheal (ET); fine particle fraction (FPF); geometric diameter (d_geom_); geometric standard deviation (GSD); Hausner Index (H); high-pressure homogenization (HPH); human bronchial epithelium cells (16-HBE); hypothalamic–pituitary–adrenal (HPA); intravenous (IV); inhaled powder (IP); Lipid–polymer hybrid nanoparticles (LPHNPs); L-leucine (LEU); mass median aerodynamic diameter (MMAD); methylprednisolone (MePD); N-acetylcysteine (NAC); Nano into Microparticles (NiM); polyamidoamine (PAMAM); polydispersity index (PDI); polymeric nanoparticles (NPs); scanning electron microscopy (SEM); simulated lung fluid (SLF4); spray drying (SD); spray-dried microparticles (MPs); tapped density (ρ_tapped_); Thin Layer Evaporation (TLE); α,β-poly(N-2-hydroxyethyl)-D,L-aspartamide (PHEA).
